# The Public Health

**Published:** 1899-09

**Authors:** W. F. Blunt

**Affiliations:** State Health Officer; Austin, Texas


### The Public Health.

State Health Officer Blunt has issued the following:

AUSTIN, TEXAS, August 1, 1899.

........................M. D.,
County Health Officer,
..................County, Texas.

DEAR DOCTOR: Article 4339, Title XCII, Revised Civil Statutes of the State of Texas, 1895, makes it your duty to make written reports to the State Health Officer whenever required to do so. It is my desire to secure complete reports from every county in the State on smallpox, cerebro-spinal meningitis and scarlet fever. It is desired that these reports embrace the period from December 1, 1898, to August 1, 1899. You will therefore please make your report on the inclosed forms as indicated. It is intended to include these reports in the forthcoming annual report of this department, giving credit to each reporter. If you are not sufficiently conversant with the facts to report your entire county, a sufficient number of blanks have been inclosed to enable you to furnish one to such physician as will be able and willing to assist you in the matter.

It is my desire to have this report from every county in Texas, and to make it valuable it is equally as necessary to have a report from counties that have *not* experienced these diseases as those that *have*.

As the law requires that I make my report to the governor on the first day of December it will be necessary to have your report to hand by the first of October so that it may be properly tabulated therein.

I am, yours very truly,

W. F. BLUNT, M. D.,
State Health Officer.

REPORT ON SMALLPOX, DIPHTHERIA AND SCARLET FEVER, for period extending from December 1, 1898, to August 1, 1899.

Number cases smallpox?

Number deaths?

How many contracted who had recently been vaccinated?

How many contracted it who have scars of former vaccination?

How many died who came under the two preceding heads?

DIPHTHERIA.

Number cases diphtheria?

Number deaths?

Number cases antitoxin was used?

Number deaths where antitoxin was used?

SCARLET FEVER.

Number cases scarlet fever?

Number deaths?

REPORT ON CEREBRO-SPINAL MENINGITIS, for the period between December 1, 1898, and August 1, 1899.

Number of cases?

Number of deaths?

Number of partial recoveries?

Complete recoveries?

Date of first case?

Date of last case?

Was there any unusual signs, symptoms or circumstances in connection with the disease?

Do you know of any cases existing in other localities prior to the first case in your county?

Was any case apparently contracted from another case, and if so, what were the circumstances?

Have you had an epidemic of influenza this year, and if so, did it precede or follow this disease?

Did you have an unusual number of cases of pneumonia, and if so, which appeared first? [?]

Did any of your cases have a "cold" or cough just preceding the disease?

